# Genomic Evolution of SARS-CoV-2 Virus in Immunocompromised Patient, Ireland

**DOI:** 10.3201/eid2709.211159

**Published:** 2021-09

**Authors:** Maureen Lynch, Guerrino Macori, Séamus Fanning, Edel O’Regan, Eoin Hunt, Dermot O’Callaghan, Brian McCullagh, Cormac Jennings, Anne Fortune

**Affiliations:** Mater Misericordiae University Hospital, Dublin, Ireland (M. Lynch, E. O’Regan, E. Hunt, D. O’Callaghan, B. McCullagh, C. Jennings, A. Fortune);; University College Dublin-Centre for Food Safety School of Public Health, Physiotherapy & Sports Science, Dublin (G. Macori, S. Fanning)

**Keywords:** 2019 novel coronavirus disease, coronavirus disease, COVID-19, severe acute respiratory syndrome coronavirus 2, SARS-CoV-2, viruses, respiratory infections, zoonoses, immunocompromised, virus evolution, B-cell therapy, superinfection

## Abstract

We examined virus genomic evolution in an immunocompromised patient with prolonged severe acute respiratory syndrome coronavirus 2 infection. Genomic sequencing revealed genetic variation during infection: 3 intrahost mutations and possible superinfection with a second strain of the virus. Prolonged infection in immunocompromised patients may lead to emergence of new virus variants.

The coronavirus disease (COVID-19) pandemic, caused by severe acute respiratory syndrome coronavirus 2 (SARS-CoV-2), has led to substantial illness and death in immunocompromised patients (*1*). Outcomes for patients with hematologic malignancies can be poor because of immune suppression associated with cancer itself and chemoimmunotherapy regimens used to treat these cancers (*2*).

Persistent shedding of SARS-CoV-2 RNA has been described since early in the pandemic; quantitative reverse transcription PCR (qRT-PCR) results have remained positive for 63 days (*3*). Recent studies of immunocompromised patients have detected infectious virus until 143 days after diagnosis (*4*–*6*). Phylogenetic analysis showed that single-nucleotide polymorphisms (SNPs) could be used to elucidate the transmission routes of SARS-CoV-2 in communities (*7*). Moreover, it has been demonstrated that intrahost single-nucleotide variants are restricted to specific lineages (*8*); however, no clear evidence supports a link between prolonged infection and intra-evolutionary dynamics (*9*). 

We report a case of a prolonged clinical infection with persistent virus shedding in a patient with functional B-cell deficiency, hypogammaglobulinemia, and COVID-19. We describe the sequence polymorphisms over time among the 9 whole-virus genome sequences obtained by following the ARTIC tiling-amplicon approach (https://artic.network/resources/ncov/ncov-amplicon-v3.pdf) and using the Illumina MiSeq platform as described (*7*).

In April 2020, a 52-year-old woman in Dublin, Ireland, sought emergency care for a 5-day history of fever, diarrhea, and fatigue. Five months earlier, she had received a diagnosis of stage 4, grade 1 follicular lymphoma and had since completed 3 cycles of chemotherapy with cyclophosphamide, vincristine, doxorubicin, prednisolone, and obinutuzumab (B-cell monoclonal antibody); the last therapy cycle had been completed 7 days before the emergency department visit. During the emergency department visit, SARS-CoV-2 was detected on a nasopharyngeal swab sample by qRT-PCR (Roche FLOW Flex, https://diagnostics.roche.com) with a cycle threshold (C_t_) value of 25.04. Chest radiographs showed a typical pattern for COVID-19 infection. The patient received hydroxychloroquine and azithromycin for 5 days. At the time of admission, she had hypogammaglobulinemia and received intravenous immunoglobulin every 4 weeks as supportive therapy.

During her 100-day hospital stay, the patient’s clinical course of illness was protracted, with fevers and oxygen requirements, requiring a 17-day stay in a critical care unit (Appendix). In the hospital, the patient was in a single room with transmission-based air-handling precautions.

During her entire hospital stay, SARS-CoV-2 was detected at varying C_t_ values in nasopharyngeal swab samples, except for days 31 and 85 when SARS-CoV-2 was not detected. Bronchoalveolar lavage (BAL) performed on day 95 to exclude other pathogens detected SARS-CoV-2 (C_t_ 30). Serologic testing did not detect antibodies to SARS-CoV-2 (Roche anti-SARS-CoV-2) on days 30, 84, and 103.

The patient was tested 17 times, and we sequenced all samples that were positive by qRT-PCR with C_t_ <32.8. All 9 samples that underwent whole-virus genome sequencing (Appendix Figure) belonged to clade 20B, lineage B.1.1. SNP analysis clustered these genomes into 3 groups. Genomes sequenced from the positive samples taken on days 5, 19, and 26 were indistinguishable at the sequence level (Figure). A sample taken on day 47 showed the first mutation event; 3 point mutations were identified in the whole-virus genome sequence data until day 76 after diagnosis. On day 82, genome analysis detected a new SNP (second mutation event). Sequencing of the BAL sample taken on day 95 detected a different set of sequence polymorphisms that most likely originated from a new infection event. SNP analysis indicated 11 point mutations (Appendix Table 1) giving rise to 3 amino acid substitutions in the gene coding for the spike protein (S:S50L, S:A653V, and S:L1186F).

**Figure:**
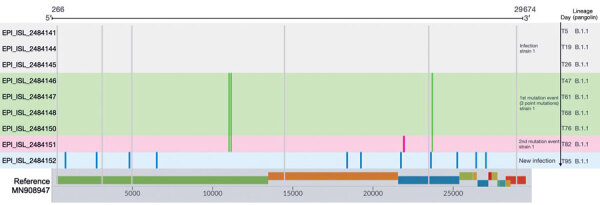
Sequence polymorphisms detected over time among the 9 whole-virus genome sequences from an immunocompromised patient with prolonged severe acute respiratory syndrome coronavirus 2 (SARS-CoV-2) infection, Ireland. The mutations are represented by different colors; gray lines indicate the polymorphisms common to the 9 whole-virus genome sequences compared with the reference whole-virus genome (GenBank accession no. MN908947, SARS-CoV-2 isolate Wuhan-Hu-1). The infection was confirmed on day 5 of infection (at admission to the emergency department), and the sequencing demonstrated stability of the virus genome sequence on days 19 (T19) and 26 (T26) after the first detection. Green indicates mutations detected in the sample at 47 days after first the emergency department admission (T47), T61, T68, and T76. At sample time T82, the strain exhibited a fourth mutation (pink) corresponding to the second mutation event. On day 95, a bronchoalveolar lavage sample from the patient was positive for SARS-CoV-2 and the whole-virus genome had a different set sequence polymorphism that probably originated from a new infection event. GISAID (https://www.gisaid.org) identification numbers are provided.

SARS-CoV-2 shedding in this patient with lymphoma, ongoing fevers, and oxygen requirements for 6 months was prolonged. The antibody-mediated ablation of B-cell precursors by B-cell directed monoclonal antibody therapy was most likely responsible for the prolonged virus shedding. This effect, combined with hypogammaglobulinemia, explains the lack of seroconversion and the protracted clinical course.

Sequential sequencing demonstrated intrahost mutations of >2 events (Figure) and accumulation of 4 SNPs. Analysis of a BAL sample taken on day 95 showed 11 point mutations giving rise to 3 aa substitutions in the gene coding for the spike protein. This observation is in accordance with findings of a recent study that detected 7 new mutations in a second virus strain in an immunocompromised patient (*10*). The BAL findings, along with ongoing symptoms, are suggestive of probable superinfection with cohabitation of 2 virus strains. However, considering that this was the only BAL sampled, we cannot exclude the possibility that the origin of this strain is the result of a different evolutionary path of the original population responsible for the first infection.

The superinfection that we describe was probably a nosocomial infection despite the transmission-based precautions taken in the patient’s single room during her hospital stay. However, no sequence data from other patients or healthcare workers on the ward could be explored to identify the source of infection.

Our report highlights the complex clinical course of SARS-CoV-2 in immunocompromised patients. This genomic analysis identified the ability of the virus to mutate and possibly coexist with another strain, resulting in superinfection in this immunocompromised patient. 

Appendix Supplemental methods and results for study of genomic evolution of severe acute respiratory syndrome coronavirus 2 in immunocompromised patient, Ireland.
