# Supplementary material for: Genomic Evolution of SARS-CoV-2 Virus in Immunocompromised Patient, Ireland
Source: Emerg Infect Dis. 2021 Sep;27(9):2499–501. doi: 10.3201/eid2709.211159 (PMC8386806; doi:10.3201/eid2709.211159)
Supplement: Appendix — Supplemental methods and results for study of genomic evolution of severe acute respiratory syndrome coronavirus 2 in immunocompromised patient, Ireland. [file 21-1159-Techapp-s1.pdf]

Article DOI: https://doi.org/10.3201/eid2709.211159

Genomic Evolution of SARS-CoV-2 Virus in Immunocompromised Patient, Ireland

Appendix

Patient's Clinical Course

A 52-year-old female presented to the Emergency Department (ED) in April 2020, with a five-day history of fever, diarrhea and fatigue. The patient was diagnosed with stage 4, grade 1 follicular lymphoma 5 months earlier, and had completed 3 cycles of chemotherapy with cyclophosphamide, vincristine, doxorubicin, prednisolone, and obinutuzamab (B-cell monoclonal antibody), the last cycle being completed 7 days before presentation to the ED. SARS-CoV-2 was detected by RT-qPCR on a nasopharyngeal swab, with a cycle threshold (C_T) value of 25.04 using the Roche flowflex platform. Chest X-ray on admission showed bilateral peripheral airspace opacities in the mid- to lower-zones with a typical pattern for COVID-19 pneumonia. The patient was treated with hydroxychloroquine and azithromycin for 5 days. On admission, the patient had hypogammaglobulinemia, and received intravenous immunoglobulin (IVIG) every 4 weeks as supportive therapy, during the hospital admission.

The patient received ward-based care for 21 days, with ongoing fevers, and was transferred to the Critical Care Unit with increasing oxygen requirements. Computed tomography (CT) of the thorax reported progressing covid pneumonitis. The patient was managed with non-invasive ventilation and self-proning. Following 17 days in Critical Care, the patient was transferred to the ward, where she remained in a single room, with transmission based precautions for the hospital stay.

In all the patient remained in hospital for 100 days, with intermittent fevers, and oxygen requirements. SARS-CoV-2 was detected in NP swabs taken during the entire admission with varying C_T values, except for days-31 and −85, when SARS-CoV-2 was not detected. A bronchoalveolar lavage (BAL) was performed on day 95, to exclude other viruses, bacteria, and fungi. SARS-CoV-2 was detected (C_T value 30) in the BAL. Serological testing did not detect

antibodies to SARS-CoV-2 (Roche Anti-SARS-CoV-2) on days 30, 84, and 103. Following discharge, the patient remained on home oxygen, with intermittent fevers until 6 months after diagnosis. She continued to receive IVIG every 4 weeks. A PET CT scan for disease staging reported good partial remission of lymphoma, and further doses of immunochemotherapy were withheld.

The decision to prolong transmission-based precautions during the hospital stay, beyond the recommended 20 days post symptom onset, was based on persistent fevers, and oxygen requirements. Repeated detection of SARS-CoV-2 in respiratory samples meant a test-based strategy could not be used, hence transmission-based precautions remained for the duration of hospital admission (*1*).

Sample preparation for sequencing bioinformatics and statistical analysis

Samples were prepared for sequencing starting from cDNA synthesis (reverse transcription) using LunaScript RT SuperMix (New England Biolabs, Ipswich, UK). The sequencing library were obtained following ARTIC tiling-amplicon approach, prepared using the NEBNext Ultra II kit (New England Biolabs, Ipswich, UK) and sequenced on an Illumina MiSeq using 300-cycle v2 reagent kits (Illumina, Cambridge, UK). Raw reads were mapped to reference sequence using Bowtie 2 (*2*) and SAMtools (*3*) was used for variants calling and generate the consensus sequences (GenBank number, MN908947.3). The read coverage was estimated using samtools flagstat tool from the SAMtools software package (*3*). The libraries generated from the nine samples yield at least 337,274 (lowest value among the 9 samples) mapped reads (primer-trimmed sequences) and the genome coverage (completion) was on average 98.34% to the reference strain (MN908947.3) for the nine samples, with values between 97.64 and 99.18% (Appendix Table 2). The average coverage for the nine samples was 4,883, with values ranging between 3,778 and 5,454 (Appendix Table 2). Comparative sequence analysis was conducted by phylogenetic inference maximum likelihood (ML) method with RAxML (*4*) using the multiple sequences aligned by MAFFT (*5*). Clades and variants were assigned respectively using Nextstrain (*6*) and Pangolin COVID-19 lineage assigner (http://pangolin.cog- uk.io/). All the sequences generated were submitted to GISAID (*7*) and the ID (gisaid_epi_isl) are presented in Appendix Table 2.

References

1. Centers for Disease Control and Prevention. Duration of isolation and precautions for adults with COVID-19. 2021 [cited 2021 May 20]. https://www.cdc.gov/coronavirus/2019-ncov/hcp/duration-isolation.html

2. Langmead B, Salzberg SL. Fast gapped-read alignment with Bowtie 2. Nat Methods. 2012;9:357–9. PubMed https://doi.org/10.1038/nmeth.1923

3. Li H, Handsaker B, Wysoker A, Fennell T, Ruan J, Homer N, et al.; 1000 Genome Project Data Processing Subgroup. The Sequence Alignment/Map format and SAMtools. Bioinformatics. 2009;25:2078–9. PubMed https://doi.org/10.1093/bioinformatics/btp352

4. Stamatakis A. RAxML version 8: a tool for phylogenetic analysis and post-analysis of large phylogenies. Bioinformatics. 2014;30:1312–3. PubMed https://doi.org/10.1093/bioinformatics/btu033

5. Katoh K, Standley DM. MAFFT multiple sequence alignment software version 7: improvements in performance and usability. Mol Biol Evol. 2013;30:772–80. PubMed https://doi.org/10.1093/molbev/mst010

6. Hadfield J, Megill C, Bell SM, Huddleston J, Potter B, Callender C, et al. Nextstrain: real-time tracking of pathogen evolution. Bioinformatics. 2018;34:4121–3. PubMed https://doi.org/10.1093/bioinformatics/bty407

7. Shu Y, McCauley J. GISAID: Global initiative on sharing all influenza data - from vision to reality. Euro Surveill. 2017;22:30494. PubMed https://doi.org/10.2807/1560-7917.ES.2017.22.13.30494

Appendix Table 1. Nucleotide mutation and amino acid substitutions in SARS-CoV-2 from immuncompromised patient with prolonged clinical infection*

	Nucleotide mutation†														
Sample	1	2	3	4	5	6	7	8	9	10	11	12	13	14	15
EPI_ISL_2484141	ND	ND	ND	ND	ND	ND	ND	ND	ND	ND	ND	ND	ND	ND	ND
EPI_ISL_2484144	ND	ND	ND	ND	ND	ND	ND	ND	ND	ND	ND	ND	ND	ND	ND
EPI_ISL_2484145	ND	ND	ND	ND	ND	ND	ND	ND	ND	ND	ND	ND	ND	ND	ND
EPI_ISL_2484146	ND	ND	ND	ND	A11054G	G11083T	ND	ND	ND	ND	ND	G23608T	ND	ND	ND
EPI_ISL_2484147	ND	ND	ND	ND	A11054G	G11083T	ND	ND	ND	ND	ND	G23608T	ND	ND	ND
EPI_ISL_2484148	ND	ND	ND	ND	A11054G	G11083T	ND	ND	ND	ND	ND	G23608T	ND	ND	ND
EPI_ISL_2484150	ND	ND	ND	ND	A11054G	G11083T	ND	ND	ND	ND	ND	G23608T	ND	ND	ND
EPI_ISL_2484151	ND	ND	ND	ND	A11054G	G11083T	ND	ND	ND	G21778A	ND	G23608T	ND	ND	ND
EPI_ISL_2484152	C678T	G2626A	G4866T	C6402T	Not present	ND	C18252T	C19264T	C21711T	ND	C23520T	ND	C25118T	C26333T	C26895T
AA Substitution‡	ORF1a: A138V	ND	ND	ORF1a: P2046L	ORF1a: S3597G	ORF1a: L3606F	ND	ORF1b: L1933F	S:S50L	ND	S:A653V	ND	S:L1186F	E:T30I	M:H125Y

*Virus isolates are denoted by a the GISAID (https://www.gisaid.org)–assigned identification number, and nucleotide polymorphisms and their positions, across the SARS-CoV-2 genome are shown. E, envelope protein; ND, not detected; M, membrane protein; ORF, open reading frame; S, spike protein; SARS-CoV-2, severe acute respiratory coronavirus 2.

†Nucleotide mutations are shown in the following format: nucleotides in the reference strain (A, adenine; C, cytosine; G, guanine; T, thymine); genomic position in the sequence; and code of the nucleotide detected in the genome of the virus sequenced.

‡Amino acid substitution at protein level are shown in the following the format: protein code (ORF1a, ORF1b, S, E and M); code of the amino acid in the reference strain (A, alanine; V, valine; P, proline; L, leucine; S, serine; G, glycine, F, phenylalanine; T, threonine; H, histidine; Y ,tyrosine); and code of the amino acid detected in the genome of the virus sequenced.

.

Appendix Table 2. The assigned identification numbers are presented along with the date of sampling the lineage as assigned by Pangolin and quality values (average coverage and % of coverage). The average coverage is calculated at the level of the single base pair and the % of coverage is the percentage of completion of the consensus sequences to the reference strain (MN908947.3)

ID*	Date sampling	Average coverage	% Coverage	Pangolin lineage
EPI_ISL_2484141	09/04/2020	5,454	99.12	B.1.1
EPI_ISL_2484144	23/04/2020	4,875	97.76	B.1.1
EPI_ISL_2484145	27/04/2020	5,446	98.31	B.1.1
EPI_ISL_2484146	21/05/2020	5,319	98.73	B.1.1
EPI_ISL_2484147	04/06/2020	5,219	99.18	B.1.1
EPI_ISL_2484148	11/06/2020	4,319	98.21	B.1.1
EPI_ISL_2484150	19/06/2020	4,570	97.74	B.1.1
EPI_ISL_2484151	25/06/2020	3,778	97.64	B.1.1
EPI_ISL_2484152	08/07/2020	4,967	98.36	B.1.1

*GISAID (https://www.gisaid.org) ID numbers.

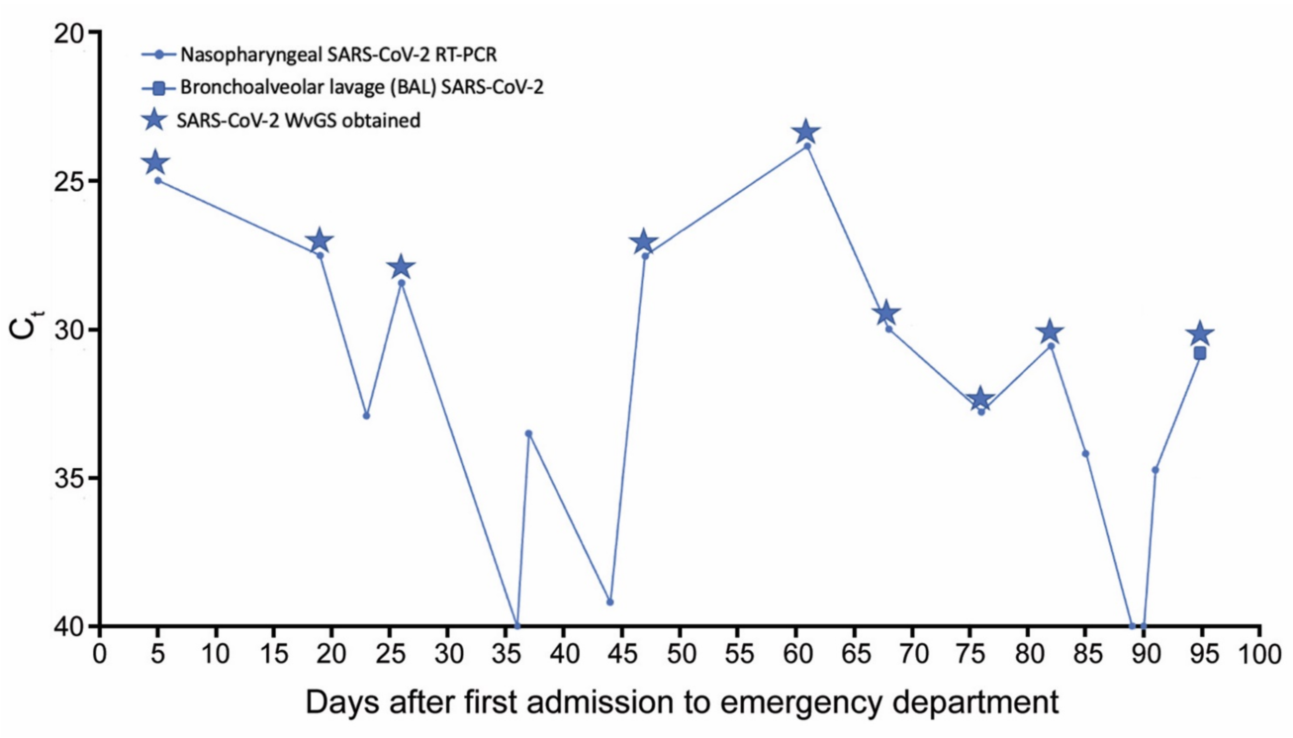

Appendix Figure. Trend of the C_T values after the first admission (T_0) to the emergency department of the patient. The points highlight the SARS-CoV-2 Whole virus Genome Sequences (WvGS) that were obtained for this study.
